# Safety and early efficacy of endoscopic sleeve gastroplasty (ESG) for obesity in a multi‐ethnic Asian population in Singapore

**DOI:** 10.1002/jgh3.12680

**Published:** 2021-12-03

**Authors:** Ravishankar Asokkumar, Chin Hong Lim, Ai Shan Tan, Phong Ching Lee, Alvin Eng, Jeremy Tan, Gontrand Lopez‐Nava, Sonali Ganguly, Jason Chang, Christopher Khor

**Affiliations:** ^1^ Department of Gastroenterology and Hepatology Singapore General Hospital Singapore Singapore; ^2^ Duke‐NUS Graduate Medical School Singapore Singapore; ^3^ Department of Upper Gastrointestinal and Bariatric Surgery Singapore General Hospital Singapore Singapore; ^4^ Department of Nutrition and Dietetics Singapore General Hospital Singapore Singapore; ^5^ Department of Endocrinology Singapore General Hospital Singapore Singapore; ^6^ Bariatric Endoscopy Unit, HM Sanchinarro Hospital Madrid Spain

**Keywords:** bariatric endoscopy, endoscopic sleeve gastroplasty, morbid obesity, obesity, overstitch, weight loss

## Abstract

**Background and Aim:**

Endoscopic sleeve gastroplasty (ESG) is an alternative nonsurgical treatment option for obesity. However, most studies on the utility and efficacy of ESG are derived from the Western population. It is unknown if ESG elicits similar results in Asians with different fat distribution, sociocultural customs, and dietary practices. Our study aims to assess the safety and efficacy of ESG among a multi‐ethnic Asian population.

**Methods:**

We reviewed 35 patient records who underwent primary ESG for obesity at our unit. We followed a U‐shaped suture pattern. Our primary outcome was to assess technical feasibility and safety. The secondary outcome was to determine the percentage total body weight loss (TBWL) at the last follow‐up.

**Results:**

The mean ± SD age and body mass index were 43.6 ± 11.3 years and 34 ± 4.9 kg/m^2^, respectively. The majority were female (57%) and of Chinese ethnicity (51%). The procedure was technically successful in all patients. We used an average of five sutures (range, 4–7), and the mean ± SD procedure time was 65 ± 10 min. No major complications occurred, and the average length of stay was 1 day. Twenty‐one patients completed 3 months of follow‐up, and 10 patients 6 months. The mean ± SD TBWL at 3 and 6 months were 14.5 ± 4.8% and 16.2 ± 4.9%, respectively. We observed improvement in diabetes mellitus (87%), fatty liver (86%), and hypertension (58%) during the follow‐up.

**Conclusion:**

ESG is a safe and effective option for promoting weight loss in a multi‐ethnic Asian population. ESG‐induced weight loss may improve obesity‐related comorbidities.

## Graphical abstract

Endoscopic sleeve gastroplasty (ESG) is an effective treatment option for Asian patients with obesity. ESG is a short‐stay procedure with an excellent safety profile, and the weight loss achieved leads to improvement in comorbidities.

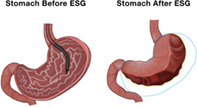

## Introduction

Obesity is a chronic, relapsing, multifactorial, and neuro‐behavioral disease associated with multiple metabolic comorbidities, including diabetes mellitus and hypertension.^1^ The prevalence of obesity and overweight among the Association of Southeast Asian Nations (ASEAN) population is steadily increasing and places a significant strain on the healthcare systems of its component nations.^2^ In Singapore, the number of obese individuals has risen by 70%, and 1 in 10 persons between 18 and 69 years of age is obese. It is projected that 14% of the population will be obese by 2024. Healthcare spending has risen exponentially, and it is estimated that US$ 400 million was spent on obesity and loss of productivity in 2016.^3^, ^4^

Several public health policies and measures have been introduced to combat the obesity pandemic. However, no single treatment is shown to cure obesity permanently and maintain weight loss over the long term. The current treatment options available for obese patients include diet and lifestyle changes, pharmacotherapy, and, the most effective, bariatric surgery. The number of patients seeking bariatric surgery has remained low despite its effectiveness. A survey conducted in 2018 by the Asia–Pacific Metabolic and Bariatric Surgery Society involving 18 Asia–Pacific countries showed a low frequency of bariatric surgery (overall, 0.057%; Singapore, 0.15%) even with a large population of eligible obese patients.^5^ The main reason for the reduced acceptance includes concerns regarding safety and risk with the procedure, side effects, cost, and irreversibility.^6^

Novel, less invasive approaches are required to overcome this disparity and to encourage more obese patients to seek treatment. Recently, endoscopic sleeve gastroplasty (ESG), a minimally invasive and reversible endoscopic procedure, was developed with a premise to reduce the size of the stomach, similar to laparoscopic sleeve gastrectomy.^7^ The available data from the West have demonstrated the procedure to be safe and effective.^8^, ^9^, ^10^, ^11^ However, there is sparse literature on its safety and utility among obese Asian patients. The body mass index (BMI) threshold for obesity among Asians (27.5 kg/m^2^) is lower than for Western populations because of the higher body fat percentage at lower BMI.^12^ Thus, more data on the safety of ESG are required. We hypothesized that ESG would be safe irrespective of BMI and ethnicity and would effectively induce weight loss. This study aims to assess the safety of ESG and its early efficacy in a multi‐ethnic cohort of patients in Singapore.

## Methods

### Trial design

We retrospectively reviewed the records of patients who underwent ESG at the Obesity Center at Singapore General Hospital between November 2020 and August 2021. The institutional review board approved the study. All authors had access to the study data and reviewed and approved the final conclusions. The study was conducted following the ethical principles detailed in the Declaration of Helsinki and was consistent with the Good Clinical Practices recommendation. Informed consent was obtained from all the patients before the procedure.

### Participants

We reviewed the records of patients who underwent primary ESG at our unit. All the patients referred for ESG had declined surgery and failed in diet and lifestyle therapy. The inclusion criteria for ESG were (i) age ≥17 years, (ii) BMI ≥27.5 kg/m^2^, and (iii) be able to comply with instructions and provide informed consent. We excluded those with: (i) severe systemic illnesses, (ii) substance abuse, (iii) uncontrolled eating disorder, (iv) pregnancy, and (v) coagulopathy. We did not pre‐select our patients, and all eligible candidates who committed to long‐term follow‐up were offered ESG. We collected information on technical outcomes, length of stay, complication rates, and early weight loss outcomes.

## Intervention

### Endoscopic sleeve gastroplasty

**Figure 1:**
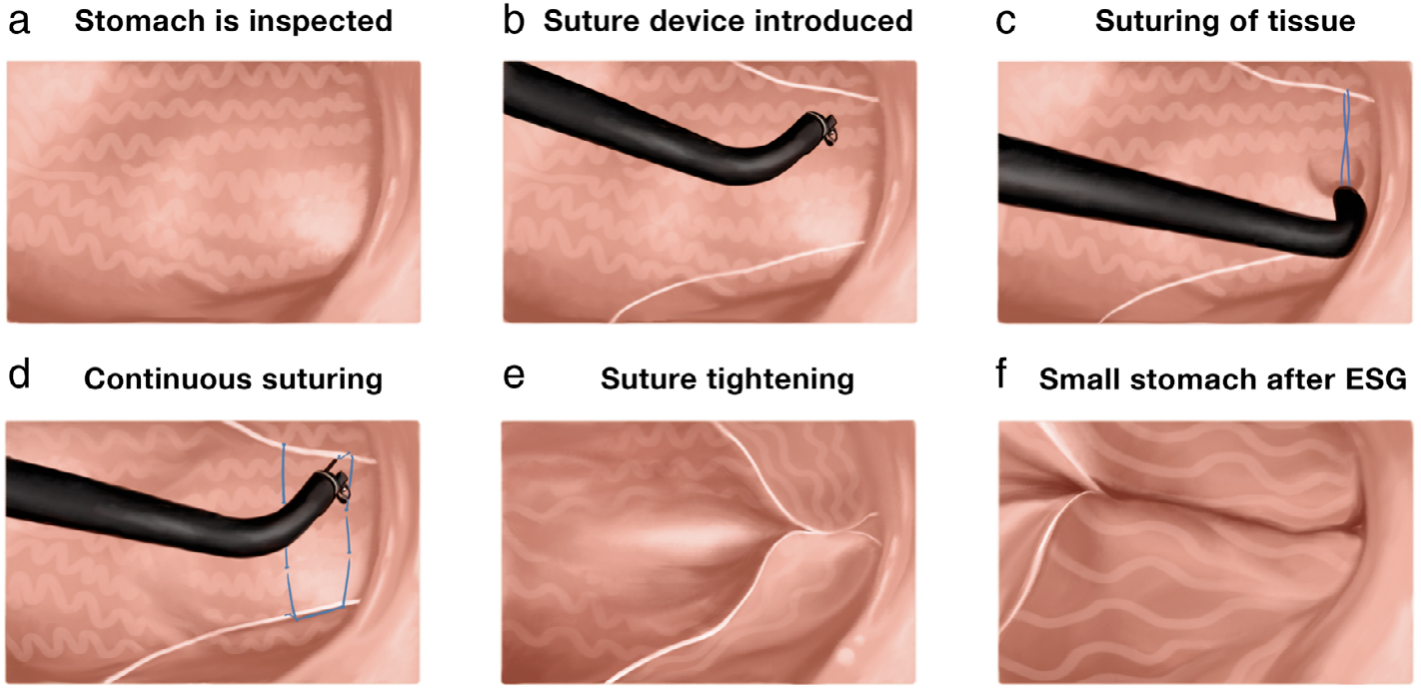
Steps of endoscopic sleeve gastroplasty and the U‐pattern of suturing. (a) Stomach is inspected; (b) the overstitch suturing device is introduced into the stomach through the mouth; (c) the tissue is captured for suturing starting at the distal body; (d) a continuous "U" shaped suturing is performed; (e) the sutured tissues are pulled together and tightened; and (f) the stomach is narrowed after ESG.

**Figure 2:**
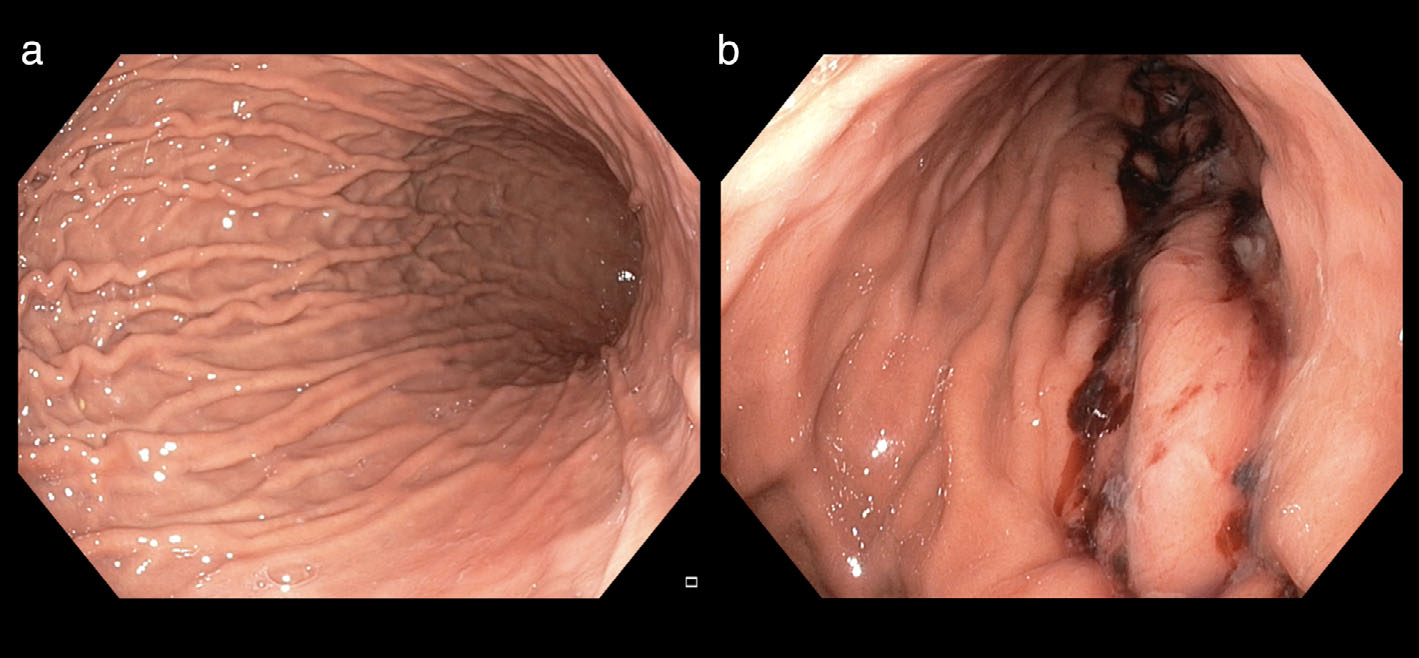
Endoscopic appearance of the stomach after ESG. (a) Normal stomach. (b) Reduced gastric volume after ESG.

All ESG procedures (Overstitch, Apollo Endosurgery, USA) were done by one endoscopist (R.A.) with extensive experience in endoscopic suturing. We performed the procedure with the patient under general anesthesia. We assessed the stomach first with an upper endoscopy to identify any contra‐indication for ESG. We then advanced the endoscope fitted with the suturing device to commence the ESG (Fig. 1). We administered antibiotics before the procedure to all the patients. We adopted a U‐shaped suture pattern starting at the distal body of the stomach and then progressed proximally, sparing the fundus.^13^ We placed 4–8 suture plications to reduce the gastric volume by approximately 70–80% (Fig. 2). We re‐evaluated the stomach after ESG to assess sleeve integrity and achieve hemostasis where required (Fig. 2). After the procedure, we monitored the patients for 24 h and then discharged them with anti‐emetics and proton pump inhibitors.

### Postprocedure follow‐up

The patients were followed up at regular intervals by dieticians, gastroenterologists, and physiotherapists. The energy requirement was calculated from the Harris–Benedict formula, taking into account the patients' physical activity status. An energy deficit of about 2.6 MJ/day was prescribed to induce an approximate loss of between 0.5 and 1 kg/week. In the first month, we maintained the patients on a strict liquid diet (4 weeks), which included commercial meal replacement options or homemade soups. We subsequently escalated intake to semi‐solid and solid food as tolerated. To prevent lean muscle mass loss, we encouraged patients to eat animal‐derived proteins such as eggs, fish, chicken, and red meat. Depending on the patient's capacity, we devised an individualized exercise plan, such as treadmill, stair‐climbing, jumping jacks, and squats (~30–45 min/day).

### Outcomes

The primary objective of this study was to assess the safety and complication rate of ESG in a multi‐ethnic Asian cohort. We graded the adverse events according to the Clavien–Dindo classification, where a higher grade represents greater severity of complication.^14^ The secondary outcome was to assess weight loss results as measured by the percentage of total body weight loss (TBWL) at the last follow‐up.

### Statistical methods

We expressed the continuous variables as mean ± SD or median (range), and categorical variables as percentages. We assessed for normality using the Shapiro–Wilk test. We used the paired *t*‐test to compare weight loss outcomes at baseline and last follow‐up after the procedure. We measured the percentage of patients achieving more than 5%, 10%, and 20% TBWL. A *P*‐value of <0.05 was considered to be significant.

## Results

### Patient characteristics

We reviewed the records of 35 patients who received ESG for obesity during the study period. The characteristics of our patients are shown in Table 1. The mean BMI was 34 ± 4.9 kg/m^2^. The mean age was 43.6 ± 11.3 years, and the initial weight was 93.2 ± 16 kg. Most of the patients were female (57%) and of Chinese ethnicity (51%). Obesity‐related comorbidities were present in 85% of patients. Six patients (17%) were on antiplatelet therapy before the procedure. We continued aspirin but stopped clopidogrel 1 week before and restarted 3 days after the procedure.

**Table 1:** Clinical characteristics of the patients

	Patients (*n* = 35)
Age ± SD, years (range)	43.6 ± 11.3
Female, *n* (%)	20 (57%)
Mean ± SD initial weight, kg	93.2 ± 16
Mean ± SD initial BMI, kg/m^2^	34 ± 4.9
Class I (≥27.5–32.4 kg/m^2^)	15 (43%)
Class II (32.5–37.4 kg/m^2^)	13 (37%)
Class III (≥37.5 kg/m^2^)	7 (20%)
Ethnicity, *n* (%)	
Chinese	18 (51%)
Indian	11 (31%)
Malay	4 (12%)
Others	2 (6%)
Comorbid illness, *n* (%)	
Hypertension	17 (49%)
Fatty liver	14 (40%)
Diabetes mellitus	8 (23%)
Coronary artery disease	7 (20%)
Liver cirrhosis	3 (8.6%)
Antiplatelets use, *n* (%)	6 (17%)
Aspirin	3 (50%)
Clopidogrel	2 (33%)
Both	1 (17%)
Completed 6 months, *n* (%)	10 (29%)
Completed 3 months, *n* (%)	21 (60%)
Completed 1 month, *n* (%)	33 (94%)
Follow‐up loss, *n* (%)	4 (11%)

### Technical outcomes and safety

The procedure was technically successful in all the patients (Table 2). We used an average of five sutures (range, 4–7). We adhered to the U‐shaped pattern in all patients. The mean procedure time was 65 min (range, 45–120 min). We did not encounter suture breakage during the procedure. The average length of stay was 24 h (range, 24–72 h). The extended hospital stay of two patients was due to patient preference and unrelated to the procedure. Postoperative recovery was uneventful in all patients. Immediate postprocedure pain occurred in 16 (46%) patients and vomiting in 8 (23%) cases. Both responded to analgesics and anti‐emetics. Nonetheless, all patients were symptom‐free at the time of discharge.

**Table 2:** Outcome of ESG in the study population

	Patients (*n* = 35)
Technical success, *n* (%)	35 (100%)
Mean no. of sutures (range)	5 (4–7)
Adherence to U‐pattern, *n* (%)	35 (100%)
Mean ± SD procedure time, min	65 ± 10.7
Mean ± SD Length of stay, days	1 ± 0.5
Postprocedure pain, *n* (%)	16 (46%)
Vomiting, *n* (%)	8 (23%)
Major complications	Nil
Mean ± SD hemoglobin change (g/dL)	0.9 ± 2.4

No major complications occurred in our cohort. In two patients, intra‐procedure bleeding was noted. The bleeding occurred at the helix puncture site in the proximal stomach. In the first case, the oozing was persistent even after cinching the tissues together. In the second case, active spurting was seen. We treated both the patients with hemoclips and adrenaline injection and achieved hemostasis. We discharged them within 24 h; no blood transfusion was required. None presented again with re‐bleeding or intractable vomiting. In our group, the average decline in hemoglobin after the procedure was 0.9 ± 2.4 g/dL.

### Weight loss outcomes

Among the study cohort, 10 (29%) patients completed 6 months of follow‐up, and 21 (60%) 3 months (Table 3). Four patients dropped out (range, 0–6 months), and the rest are in the early follow‐up phase. The overall mean ± SD TBWL, %TBWL, and BMI loss at 3 months was 13.2 ± 4.8 kg, 14.5 ± 4.8%, and 5.1 ± 1.6 kg/m^2^, respectively. Similarly, the mean ± SD TBWL, %TBWL, and BMI loss at 6 months was 14.1 ± 5.9 kg, 16.2 ± 4.9%, and 5.7 ± 1.5 kg/m^2^, respectively.

**Table 3:** Weight loss outcome after ESG

Outcomes	1 month	3 months	6 months	*P*‐value
Mean ± SD TBWL, kg	8.6 ± 2.8	13.2 ± 4.8	14.1 ± 5.9	<0.001
Mean ± SD %TBWL	9.4 ± 2.8	14.5 ± 4.8	16.2 ± 4.9	<0.001
Mean ± SD ΔBMI, kg/m^2^	−3.2 ± 1.1	−5.1 ± 1.6	−5.7 ± 1.5	<0.001

Pairwise comparison showed no difference in weight loss between 3 and 6 months.

Among the study cohort, more than 5%, 10%, 15%, and 20% TBWL was achieved in 94%, 74%, 32%, and 17%, respectively. Among the four patients who dropped out, two achieved >10% TBWL. The other two cases, despite multiple reminders, never returned for appointments after the procedure.

### Comorbidity status

We observed improvement in comorbidities within the short follow‐up duration. Among the patients with type 2 diabetes (*n* = 8), we observed improvement in HbA1c or reduction in medication dosage in seven (87%) patients. The decline in HbA1c, compared to baseline, was seen within the first 3 months of follow‐up post ESG (8 ± 0.9% *vs* 7 ± 0.9%). In six (75%) patients, the dosage of the diabetic drugs was reduced, and in two (25%) patients insulin was stopped completely. A decrease in the pill burden was noticed at 3 months after ESG (median; 3 *vs* 2). Likewise, elevated transaminases and fatty liver were seen in 14 patients at baseline. We observed improvement in liver tests in 12 (86%) patients. ESG, as compared to baseline, resulted in remarkable improvement in ALT (68.2 ± 38.2 *vs* 30 ± 11.9 IU/L) and AST (54.6 ± 27.4 *vs* 32.8 ± 7.4 IU/L), respectively. In 10 of the 17 cases (59%), we observed improvement in systolic blood pressure and medication reduction for hypertension. The mean difference in the systolic blood pressure was 7.7 mmHg. In two cases, all the medications were stopped altogether.

## Discussion

Our study showed that ESG was safe in a Southeast Asian cohort irrespective of their BMI. There were no severe complications. All the patients recovered rapidly and required only an overnight hospital stay. ESG induced significant weight loss in our patients, albeit over a short follow‐up duration, resulting in improvement in obesity‐related comorbidities.

Endoscopic suturing in bariatrics was first introduced in 2004, and since then significant advancement has occurred.^15^ The ESG procedure was developed with the intent to induce gastric restriction, similar to laparoscopic sleeve gastrectomy. Over the years, the technique has undergone several changes and refinement. In earlier studies, the procedure was performed in retroflexion, targeting the fundus first and then progressing to the antrum.^16^ However, this was complex and technically challenging. Besides, many suture materials (23–38) and different suture patterns, such as interrupted sutures, M pattern, Z pattern, and triangular pattern, were used to achieve gastric restriction. Currently, we perform all the ESG procedures in anteflexion and spare the fundus. The intact fundus functions like a reservoir retaining the meal to cause a sensation of fullness.^17^ A recent study comparing suturing *versus* no suturing of the gastric fundus in ESG found no added benefit and lower efficacy with fundal suturing.^18^ We utilized the U‐pattern in all cases. Our previous analysis comparing different suture patterns from international registry data showed superior results with the U‐pattern.^19^

Multiple studies primarily involving mainly the Western population have established the safety and efficacy of ESG. Hedjoudje *et al*., in a meta‐analysis involving 1772 ESG patients, showed the pooled rate of adverse events to be 2.2%.^20^ This is significantly lower than the value reported with LSG (11.8%).^10^ In our cohort, no patients developed serious adverse events requiring additional endoscopic or surgical intervention after ESG. Among the patients receiving antiplatelet agents, none developed intra‐ or postprocedural bleeding. Our postprocedure blood analysis did not show a significant decline in hemoglobin. One concern with gastric volume reduction following LSG is the development of de novo reflux disease and Barrett's esophagus.^21^ None of our patients reported reflux symptoms or required initiation of proton pump inhibitors during the follow‐up. In ESG, the anatomy as well as vascular and neural innervation of the stomach is preserved. Additionally, the angle of His and fundus is left intact, and the functional barrier to acid reflux is maintained.

ESG delivers its weight loss effect predominantly by achieving gastric restriction and altering gastric motility.^22^ The multiple continuous full‐thickness plications reduce gastric volume by 75–80% and restrict gastric accommodation and emptying. Abu Dayyeh *et al*. demonstrated that ESG prolonged gastric emptying of solids by 90 min and resulted in a 59% decrease in caloric intake.^22^, ^23^, ^24^ A majority (90%) of our patients reported a subjective reduction in meal volume during follow‐up and attained early satiation following a meal. ESG is also postulated to generate weight‐loss‐dependent changes in gut hormones. We compared gut and metabolic hormone changes after ESG at 6 months in our previous study.^25^ We demonstrated that the ghrelin levels, which usually rise following weight loss, remained unaltered after ESG, thereby supporting the influence of ESG on the hunger pathway. Likewise, there was a shift in the insulin secretory pattern, improvement in insulin resistance, and a profound lowering of leptin levels. In our cohort, we observed improvement in diabetes mellitus and fatty liver disease in most patients by 3 months, favoring ESG's role in improving insulin resistance.

A weight loss of 5–15% improves all obesity‐related comorbidities, enhances the quality of life, and decreases mortality.^26^ ESG has consistently been shown to achieve the weight loss required to demonstrate comorbid improvement similar to LSG in the short term.^27^ A study comparing ESG with LSG demonstrated a TBWL of 18.5% with ESG compared to 28.3% with LSG at 2 years.^28^ Sharaiha *et al*. showed that the weight loss achieved could be maintained for 5 years with a good multidisciplinary follow‐up.^29^ The early weight loss results observed in our cohort appear to follow a similar trend. Although ESG induces significant weight loss, the quantum of weight loss is lower than with bariatric surgery. However, with reduced uptake of bariatric surgery among patients with obesity, the availability of such minimally invasive treatment may encourage more individuals to seek treatment and serve as a bridge to surgery, especially in Asia, where surgery is often less desired.^30^, ^31^

Our study has several strengths and certain limitations. We present the first experience investigating the safety and utility of ESG in a relatively large group of multi‐ethnic Asian patients. It is essential and relevant to show such results, as different patient populations might respond variably to weight loss treatment. Additionally, the dietary habits, physical activity pattern, sociocultural beliefs, and outlook toward obesity management diverge significantly between the East and the West.^32^ One challenge we encountered was patients' expectations and desire to see results instantly, rather than approaching weight management as a long‐term treatment. It is therefore critical to deliver such therapy within a multidisciplinary setting. Our patients were counseled before their ESG procedure and followed up by a multidisciplinary team (MDT) with expertise in obesity management. All the procedures were performed by the same endoscopist with extensive experience (>100 procedures) in ESG. There was no variation in technique or postprocedure instructions. There is a learning curve to achieve mastery in the technique. The available evidence indicates that this mastery in ESG is achieved after 55 procedures.^33^ The replicability of similar safety and efficacy with ESG in other Asian centers requires further study. Our study is limited by its retrospective design, short follow‐up, and lack of a control group. We included all the consecutive patients who underwent ESG, and there was no selection bias. The weight loss and safety data were recorded prospectively in our database. We are actively monitoring the patients to assess their long‐term outcomes. Most of the patients who opted for ESG have failed diet and lifestyle therapy. The available evidence has shown ESG to be superior to conventional treatment. Our main objective was to report the safety profile of ESG and patient experience in this population. We noticed early follow‐up loss in some patients similar to that observed in our bariatric surgery patients.^34^ Rigorous patient selection and establishing a commitment to MDT follow‐up are critical to prevent follow‐up loss. We anticipated even greater weight loss; however, ongoing COVID‐19 pandemic restrictions limited participation in physical activity sessions and regular follow‐up attendance. Despite these limitations, most of our patients achieved weight loss that was sufficient to effect improvement in their comorbidities.

In conclusion, ESG delivers acceptable early weight loss results with an excellent safety profile in this cohort of Southeast Asian patients with obesity. The expectation of rapid recovery and the reversible and repeatable nature of the procedure may encourage patients to seek treatment, including those who decline or are not suitable for bariatric surgery. Future studies of durability, long‐term efficacy, and cost effectiveness of ESG are required.
